# Cross Talk between KGF and KITLG Proteins Implicated with Ovarian Folliculogenesis in Buffalo *Bubalus bubalis*

**DOI:** 10.1371/journal.pone.0127993

**Published:** 2015-06-17

**Authors:** Deepak Panwar, Leena Rawal, Neeta Sehgal, Sher Ali

**Affiliations:** 1 Molecular Genetics Laboratory, National Institute of Immunology, Aruna Asaf Ali Marg, New Delhi-110067, India; 2 Department of Zoology, University of Delhi, Delhi- 110007, India; Russian Academy of Sciences, Institute for Biological Instrumentation, RUSSIAN FEDERATION

## Abstract

Molecular interactions between mesenchymal-derived Keratinocyte growth factor (KGF) and Kit ligand (KITLG) are essential for follicular development. These factors are expressed by theca and granulosa cells. We determined full length coding sequence of buffalo KGF and KITLG proteins having 194 and 274 amino acids, respectively. The recombinant KGF and KITLG proteins were solubilized in 10 mM Tris, pH 7.5 and 50 mM Tris, pH 7.4 and purified using Ni-NTA column and GST affinity chromatography, respectively. The purity and molecular weight of His-KGF (~23 kDa) and GST-KITLG (~57 kDa) proteins were confirmed by SDS-PAGE and western blotting. The co-immunoprecipitation assay accompanied with computational analysis demonstrated the interaction between KGF and KITLG proteins. We deduced 3D structures of the candidate proteins and assessed their binding based on protein docking. In the process, KGF specific residues, Lys123, Glu135, Lys140, Lys155 and Trp156 and KITLG specific ones, Ser226, Phe233, Gly234, Ala235, Phe236, Trp238 and Lys239 involved in the formation of KGF-KITLG complex were detected. The hydrophobic interactions surrounding KGF-KITLG complex affirmed their binding affinity and stability to the interacting interface. Additionally, *in-silico* site directed mutagenesis enabled the assessment of changes that occurred in the binding energies of mutated KGF-KITLG protein complex. Our results demonstrate that in the presence of KITLG, KGF mimics its native binding mode suggesting all the KGF residues are specific to their binding complex. This study provides an insight on the critical amino acid residues participating in buffalo ovarian folliculogenesis.

## Introduction

Protein-protein interactions are necessary for almost all the biological functions. The role of a protein and its interaction with other protein is determined by its 3D structure that allows fathoming active residues involved in binding, nature of its interface and conformational changes adopted by the protein. It has been reported that molecular interactions between theca and granulosa cells are important for ovarian folliculogenesis marked by exponential expansion and differentiation of the granulosa cells and maturation of the oocyte [1]. This event is regulated by both endocrine and intraovarian mechanisms, coordinating the processes of oocyte growth and differentiation. Paracrine interactions between the oocyte and surrounding granulosa cells are crucial for follicular cell development, regulated by the interplay of various hormonal factors such as neuropeptides and cytokines. However, the mechanisms of action of these factors involved in ovarian folliculogenesis are not yet fully explored [1–3].

Keratinocyte growth factor (KGF) expressed by mesenchymal theca cells is a paracrine growth and differentiation factor, belonging to heparin-binding FGF family with a distinctive pattern of target-cell specificity. KGF acts by binding with KGFR/FGFR2IIIb receptor, a splice variant of the FGF receptor 2 (FGFR2) that is predominantly expressed in the granulosa cells of growing follicles [4–6]. It is known to accentuate epithelial cell proliferation, differentiation and migration besides DNA synthesis [4,7,8]. KGF promotes growth of primordial as well as secondary follicles and reduces apoptosis of granulosa cells and preantral/preovulatory follicles [9,10,11].

On the other hand, Kit ligand (KITLG) expressing in the granulosa cells has received considerable attention for its indispensable roles in mammalian folliculogenesis, gametogenesis and hematopoiesis [12]. Its biological effects encompass binding and activation of a tyrosine kinase receptor designated as C-kit or stem cell factor (SCF) receptor, present in the oocyte and theca cells [13,14]. KITLG localized in the oocytes during all the stages of follicular development stimulates theca cell growth in the bovine ovary [15–18].

Molecular interactions between theca and granulosa cells are essential for follicular development in the ovary. KGF and KITLG are known to interact and play an important role in the mesenchymal-epithelial communication essential for folliculogenesis [8]. Such favorable interactions between KGF and KITLG have been demonstrated in rat and cattle [10,19]. However, the nature of interactions varies across the species. Thus far, no reports on the buffalo KGF and KITLG structural analysis and interacting/binding residues are available. We used bioinformatics and structure oriented approach to determine the amino acids engaged in KGF and KITLG interaction. In the process, we elucidated the KGF-KITLG binding sites involved in ovarian folliculogenesis bringing the same closer to reproductive pathways and infertility related issues, if any.

## Materials and Methods

### Sample collection

Buffalo ovaries were obtained from the Gazipur slaughter house, Delhi, India following strictly the Institute’s Ethical and Biosafety guidelines and due approvals were taken from these committees. Following this, any additional approvals for the present study were not required. The samples were frozen in liquid nitrogen and stored at −80°C until total RNA extraction was done.

### RNA isolation and cDNA synthesis

Total RNA was isolated from the ovary following standard protocols and stored at—80°C, quality and integrity of RNA was tested on 1% formaldehyde agarose gel [20]. Further, RNA was quantified using a NanoDrop (ND-1000 Spectrophotometer, Thermo Fisher Scientific, USA) and tested for genomic DNA presence with *β-actin* primers (*ACTB*) (forward: 5'CAGATCATGTTCGAGACCTTCAA3' and reverse: 5'GATGATCTTGATCTTCATTGTGCTG3'). Following this, cDNA was synthesized using cDNA RT kit (Applied Biosystems, USA). The success of cDNA synthesis was confirmed by PCR amplification with a set of bubaline derived *β-actin* primers [21].

### Cloning and isolation of full length coding sequence of *KGF* and *KITLG* genes

*KGF* and *KITLG* genes were amplified by end point PCR using ovary cDNA as template and set of primers based on *Bubalus bubalis KGF* (GenBank accession no. KP284165) and *KITLG* (GenBank accession no. KP284166) coding sequence with overhangs containing restriction sites for *Xho*I*/Nco*I and *Bam*HI/*Xho*I, respectively (Table 1). The PCR reaction conditions involved initial denaturation at 95°C for 3 minutes, followed by 35 cycles each with a subsequent denaturation at 95°C for 1 minute, annealing at 52.0°C (*KGF*) and 54.0°C (*KITLG*) for 1.5 minutes and extension at 72°C for 2 minutes followed by a final extension at 72°C for 10 minutes. Each PCR product was electrophoresed on 1% agarose gel in 1x TAE buffer, sliced and DNA was eluted using gel extraction kit (Qiagen, Germany) [20]. Subsequently, eluted DNA fragments corresponding to *KGF* and *KITLG* were cloned between the respective restriction enzyme sites of His-tagged pET28a (Novagen, USA) and GST-tagged pGEX-4T1 (Amersham Bioscience) vectors, respectively. The positive clones screened by colony-PCR and restriction digestion were then sequenced on Applied Biosystems 3130xl genetic analyzer on a 50 cm, 16 capillary array using BigDye Terminator v3.1 cycle sequencing kits (Applied Biosystems, Foster city, CA, USA) employing standard protocols [22].

**Table 1:** Details of primers used for amplification of full length coding sequence of *KGF* and *KITLG*.

Genes	Primer Sequence*	Restriction Site	Amplicon Size (bp)
*KGF*	Frd 5’-CCG*CTCGAG*ATGCGCAAATGGATACTGA-3’	*Xho*I	585
	Rev 5’- CCTATGGCAATAACCTAA*CCATGG*CATG-3’	*Nco*I	
*KITLG*	Frd 5’- CGC*GGATCC*ATGAAGAAGACACAAACTT-3’	*Bam*HI	825
	Rev 5’- AGAGTTTCAAGAAGTGTAA*CTCGAG*CGG-3’	*Xho*I	

*The restriction sites in the respective primer sets have been italicized and underlined.

### Expression and purification of recombinant KGF and KITLG proteins

The 6x-His tagged plasmid (pET28-His-KGF) was used to transform BL21 (DE3) (Stratagene) strain of *Escherichia coli* grown in LB media containing 50 μg/ml kanamycin until an OD_600_ of 0.6 at 37°C was achieved. The protein expression was induced using 1 mM isopropyl 1-thio-β-d-galactopyranoside at 37°C for 4hrs. Subsequently, cells were harvested by centrifugation at 6000 rpm for 10 minutes at 4°C, resuspended in lysis buffer (50 mM Tris, pH 7.5, 100 mM NaCl and 8 M urea) and sonicated for 8 cycles of 1 minute each. Supernatant containing KGF protein was incubated with nickel-nitrilotriacetic acid-agarose (Qiagen, USA) with end-to-end shaking for 7 hours at 4°C and the protein was eluted in elution buffer (50 mM Tris pH 7.5 and 100 mM NaCl) containing 50–250 mM imidazole. Following this, chromatography fractions containing His-KGF were dialyzed against buffer containing 10 mM Tris, pH 7.5.

For expression of GST-KITLG, the plasmid DNA was used to transform *Escherichia coli* BL21-RIL (Stratagene) strain and cells were harvested in LB media containing 100 μg/ml ampicillin and 30 μg/ml chloramphenicol. The protein expression was induced by incubation of cells with 1 mM isopropyl 1-thio-β-d-galactopyranoside at 37°C for 6 hours. Following this, cell pellets were suspended in ice-cold lysis buffer, containing 50 mM Tris, pH 7.4, 2 mM EDTA, 1 mM dithiothreitol, 1% Triton X-100, and proteases inhibitors (1 mm phenylmethylsulfonyl fluoride, 10 μg/ml leupeptin and 10 μg/ml pepstatin), and sonication was carried for 6 cycles of 1 minute each. The resultant cell debris was removed by centrifugation at 13,000 rpm for 40 minutes at 4°C. The protein from the cell lysates were affinity-purified using glutathione-sepharose resin [23]. Thereafter, the resin was washed with lysis buffer, and bound proteins were eluted with 50 mM Tris, pH 6.5, with 10 mM glutathione. Finally, purified proteins were dialyzed against 50 mM Tris, pH 7.4, 1 mM dithiothreitol and 10% glycerol. The concentration for both the purified KGF and KITLG proteins were assessed using Bradford assay.

### SDS-PAGE and Western blotting

Chromatography fractions and purified proteins were electrophoresed on SDS-PAGE and visualized by Coomassie Brilliant Blue R-250 (Sigma Aldrich, USA). For western blotting, purified proteins were subjected to 15% (w/v) SDS-PAGE and electro transferred onto nitrocellulose membrane (Bio-Rad Laboratories, CA, USA). Membranes were blocked with 5% bovine serum albumin (Sigma Aldrich, USA) in PBST (1X PBS in 0.05% Tween-20) for 2 hours at room temperature and incubated overnight with diluted anti-His and anti-GST antibodies (Sigma Aldrich, USA) in blocking buffer (1:1000). The blots were washed with PBST and incubated with horseradish peroxidase (HRP) conjugated anti-rabbit IgG (Sigma Aldrich, USA) diluted in PBST (1:10,000). After subsequent washing, the blots were developed with 1 mg/ml 3,3’-diaminobenzidine (DAB) (Sigma Aldrich, USA) and 1 μl/ml hydrogen peroxide in phosphate buffer saline.

### Co-immunoprecipitation

Total cellular ovary tissue extract was prepared by homogenizing in RIPA buffer (50 mM Tris pH 7.6, 150 mM sodium chloride, 1% Na-deoxycholate, 0.1% SDS and 1% NP-40) and protease inhibitor cocktail followed by a constant end-to-end rotation for 2 hours at 4°C. The extract was then centrifuged at 10,000 rpm for 15 minutes at 4°C. The supernatant containing the protein was collected. The total protein concentration was determined using Bradford assay (Thermo scientific, USA). Approximately 300–500 μg of protein lysate was incubated with ∼1 μg of anti-KGF IgG (Santa Cruz, USA) for 12 hours at 4°C on an end-to-end shaker in a 200 μl reaction volume. Subsequently, 50 μl of protein A+G Sepharose (Amersham-Pharmacia Biotech) was incubated with the antigen-antibody protein complex for 4–6 hours at 4°C with end to end shaking. The complex was washed thrice with RIPA buffer and eluted in 30 μl sample buffer containing reducing agent. It was then boiled at 95°C for 10 minutes and separated on 15% SDS-PAGE (w/v). Co-immunoprecipitation of KGF-KITLG complex was ascertained employing western blotting (mentioned earlier) using anti-KITLG IgG (1:1000, Santa Cruz, USA) as primary antibody and HRP conjugated anti-goat IgG as secondary antibody (1:10,000, Sigma Aldrich, USA). In a reciprocal manner, we immunoprecipitated with anti-KITLG antibody, followed by western blotting using anti-KGF IgG. The blots were developed and specific bands were observed with ECL detection system (Thermo scientific, USA).

### *In silico* sequence analysis

The putative identity of *KGF* and *KITLG* both at nucleotide and protein levels was ascertained using the BLAST program (http://blast.ncbi.nlm.nih.gov/Blast.cgi). The open reading frame (ORF) for the candidate proteins was obtained using the ORF finder (http://www.ncbi.nlm.nih.gov/gorf/gorf.html). Nucleotide sequences representing complete ORF were translated into protein sequence using ExPASy translation tool (http://web.expasy.org/translate/) and used for *in silico* characterization. Homologous conserved domains were identified with Conserved Domain Database (CDD) and secondary structures for both KGF and KITLG proteins were built based on the predictions obtained from SOPMA [24,25].

### Protein 3D structure prediction

To infer the homologous protein structures for an appropriate template based on the maximum identity and lower e-value, KGF sequence was subjected to PSI-BLAST against Protein Data Bank (PDB) [26]. The three dimensional structure of *Rattus norvegicus* (PDB ID: 1QQK Chain A, Resolution: 3.10 Å) with 97% identity was used as template for homology modeling of buffalo KGF protein using MODELLER(V9.14) [27,28]. Five models generated were ranked based on their normalized discrete optimized protein energy (DOPE) scores. The model with the lowest DOPE score was considered as the best and further optimized with its C^α^ RMSD (root-mean-square deviation) value to its template upon superposition. The protein model of KITLG was generated using Iterative Threading Assembly Refinement (I-TASSER) server (http://zhanglab.ccmb.med.umich.edu/I-TASSER/) [29]. The best predicted model for KITLG was selected on the basis of threading sequence identity and confidence score (C-score).

### Energy minimization and evaluation

3D models of the candidate proteins were subjected to structural refinement and energy minimization using YASARA force field in YASARA energy minimization server, without fixing any atoms [30]. The stereochemical qualities for energy minimized models were discerned through Ramachandran pot using PROCHECK [31]. The WHATIF server confirms the average coarse packing qualities and Ramachandran Z-scores of the refined structures [32]. Non-bonded interactions among different atoms of models were validated using ERRAT [33]. X-ray and NMR spectroscopic structural validation were verified by ProSA-web server [34].

### Assessment of the binding site residues and protein docking

Active binding site residues of KGF and KITLG protein were identified using Computed Atlas of Surface Topography of protein (CASTp) server [35]. CASTp identifies active site residues and measures volume of pockets and cavities within the 3D model. To investigate the interactions between KGF and KITLG, docked complexes were generated with the HADDOCK server [36]. As the NMR and mutagenesis data were unavailable, the passive residues were not defined between KGF and KITLG proteins. Therefore, the binding residues predicted by CASTp were used to generate ambiguous interaction restraints (AIRs) using the HADDOCK server. These restraints were combined in multidocking to generate KGF-KITLG docked complex. Rigid-body docking by HADDOCK generated 1000 models at the first iteration. Following this, best 200 structures were selected (based on energy), and then allowed to perform a second iteration semiflexible simulated annealing protocol [37]. Further, structures were refined in explicit solvent and clustered according to HADDOCK score. Upon cluster-structural analysis, 10 lowest energy models were selected, and among these, the best one was characterized on the basis of lowest HADDOCK score, electrostatic energy and Z-score [38,39].

### Protein interface and *in-silico* mutagenesis

PISA (Protein Interfaces, Surfaces, and Assemblies) was used to analyze the protein-protein interactions and binding interface of KGF-KITLG docked complex (http://www.ebi.ac.uk/pdbe/prot_int/pistart.html) [40]. The hydrophobic interaction network across the binding interface was generated using DIMPLOT (assessed using LigPlot^+^ v.4.5.3 software) [41]. Default variables were used for determining hydrogen bonds and hydrophobic interactions. The output was given in a postscript format designating all interacting residues residing between the two candidate proteins. Additionally, *in-silico* mutagenesis was performed by mutating the binding residues of KGF protein participating in the KGF-KITLG interaction using PyMOL software so that the overall electronic nature of the side chains remains unchanged [42]. The generated KGF mutant models were docked individually with the native KITLG structure and their interactions energies were assessed using PISA program.

### Homology based binding interface prediction

A significant homology exists between the KGF and KITLG protein sequences in buffalo and cattle, belonging to bovidae family. Therefore, to decipher the status of binding interface of buffalo KGF-KITLG complex across its homologs, similar docking studies were performed with that of cattle. The generated structures were visualized using PyMOL viewer (https://www.pymol.org/).

## Results

### Expression and purification of recombinant KGF and KITLG proteins

**Fig 1:**
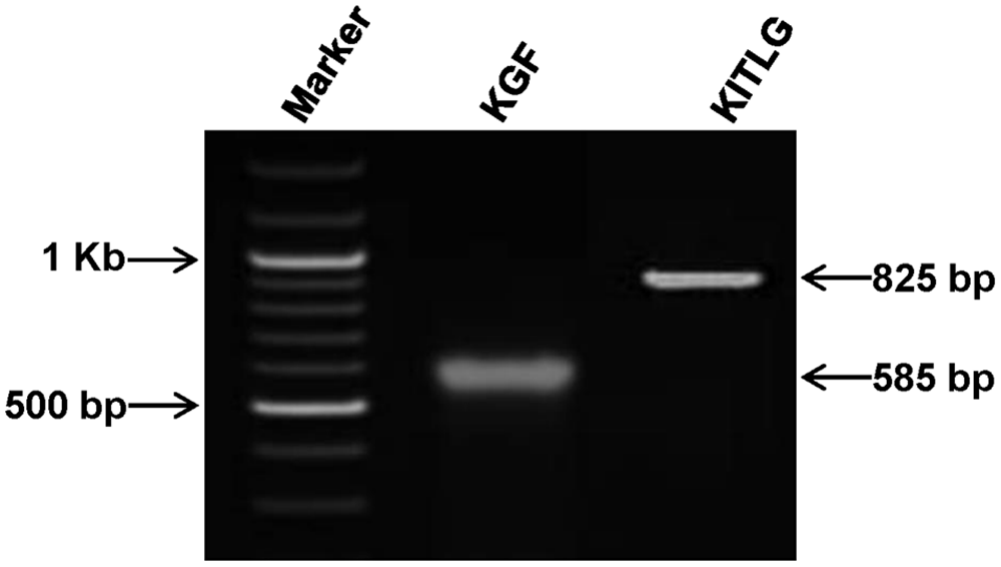
*Bubalus bubalis KGF* and *KITLG* genes amplification. Representative gel picture showing the amplicons corresponding to *KGF* and *KITLG* genes. For size marker, 100 bp ladder was used.

**Fig 2:**
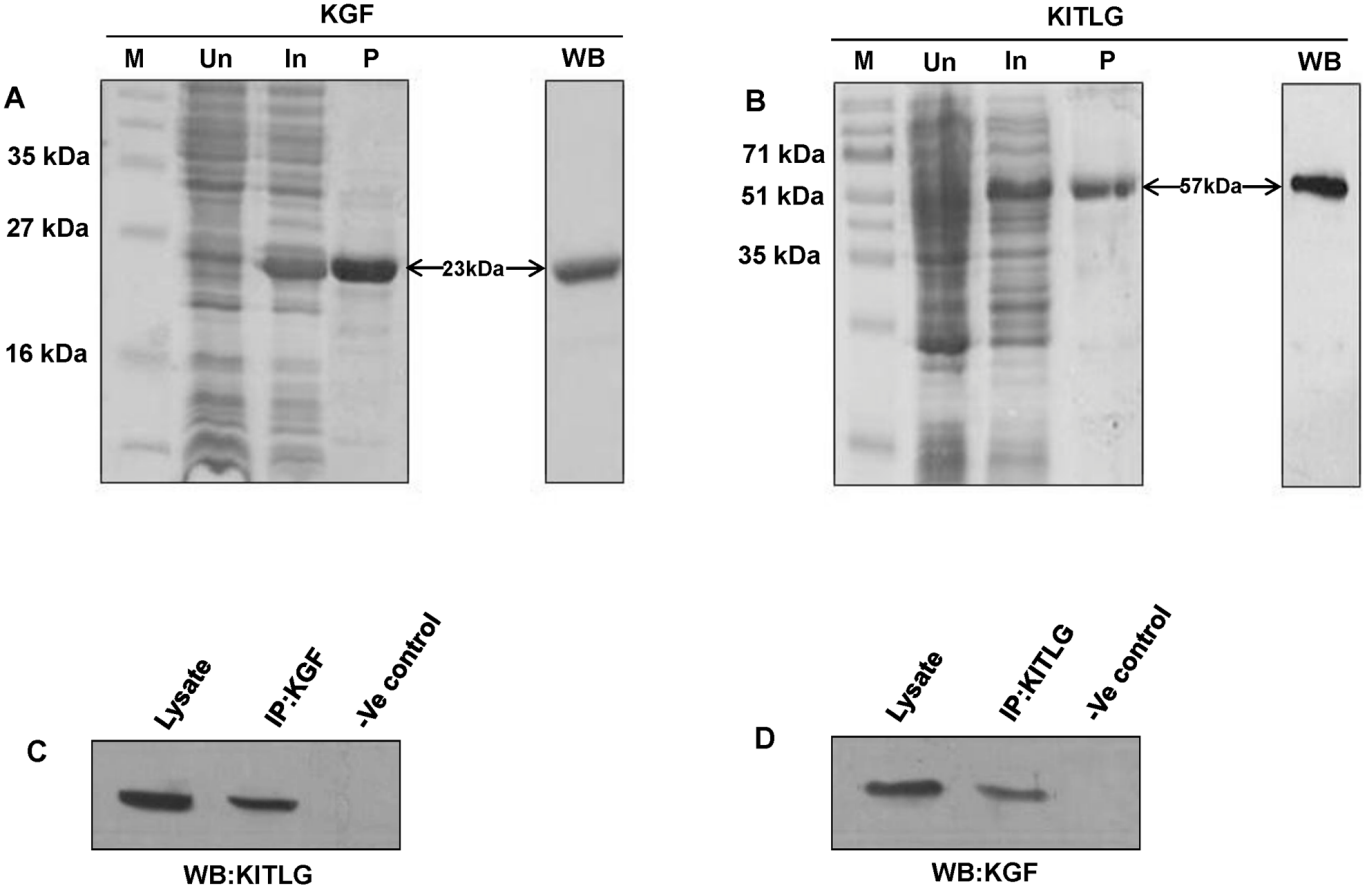
SDS-PAGE and validation of buffalo KGF and KITLG proteins. SDS-PAGE profiles of buffalo KGF (A) and KITLG (B) proteins showing their resolved chromatographic fractions. M, Un, In and P denote marker, uninduced, induced and purified protein samples. The purified KGF and KITLG proteins were validated using western blot (WB) with anti-His (panel A) and anti-GST antibodies (panel B), respectively. The 23 and 57 kDa bands correspond to the purified KGF (His-tagged) and KITLG (GST tagged) proteins. (C and D) Co-immunoprecipitation of KGF and KITLG proteins. (C) The buffalo KGF and KITLG protein interactions were confirmed by immunoprecipitation of the tissue (ovary) lysate with anti-KGF antibody followed by immunoblotting with anti-KITLG IgG and detected a specific band of 31 kDa corresponding to KITLG protein. (D) In the reciprocal assay, a band of 22 kDa was detected on immunoprecipitation with anti-KITLG antibody followed by western blotting with anti-KGF IgG. The tissue lysate and resin in both C and D panels denote the positive and negative controls, respectively.

The amplification of the PCR products corresponding to *Bubalus bubalis KGF* and *KITLG* are shown in Fig 1. The full length coding sequence of *KGF* and *KITLG* were deduced to be of 585 bp and 825 bp, respectively. Accordingly, *in silico* analysis showed the presence of 194 and 274 amino acids corresponding to KGF and KITLG proteins, respectively. The recombinant His-KGF and GST-KITLG proteins were purified using immobilized metal and glutathione affinity chromatography techniques, respectively. The SDS-PAGE and western blot analysis showed purified KGF (His-tagged) and KITLG (GST-tagged) proteins with bands corresponding to 23 and 57 kDa, respectively, thus, in accordance with their theoretical molecular weights (Fig 2A and 2B).

### *In-vivo* interaction between KGF and KITLG proteins

The KGF-KITLG interaction was confirmed by immunoblotting the antigen-antibody (lysate + anti-KGF IgG) complex using anti-KITLG antibody and detected a specific band of 31 kDa coinciding with the molecular weight of KITLG protein (Fig 2C). Further, reciprocal co-immunoprecipitation assay confirmed their interaction by immunoprecipitation with anti-KITLG antibody followed by western blotting using anti-KGF, which showed a 22 kDa band, in accordance with the KGF protein molecular weight (Fig 2D). These observations validated the *in-vivo* interaction between KGF-KITLG proteins that indeed result in a complex formation.

### Secondary structure and conserved domain

KGF was found to have 15.98%, 8.76%, 20.62% and 54.64% of α helices, β turns extended strands and random coils, respectively. A stretch of 125 residues (ranging from position 67–191) in KGF protein belonged to FGF superfamily domain, suggesting its active participation in patterning and differentiation during vertebrate embryogenesis. Whereas, KITLG encompasses 51.46% α helices, 6.20% extended strand, 2.92% β turns and 39.42% random coils. The amino acids residing from 1–274 position in KITLG protein belonged to stem cell factor (SCF) superfamily domain.

### 3D structure modeling and validation

**Fig 3:**
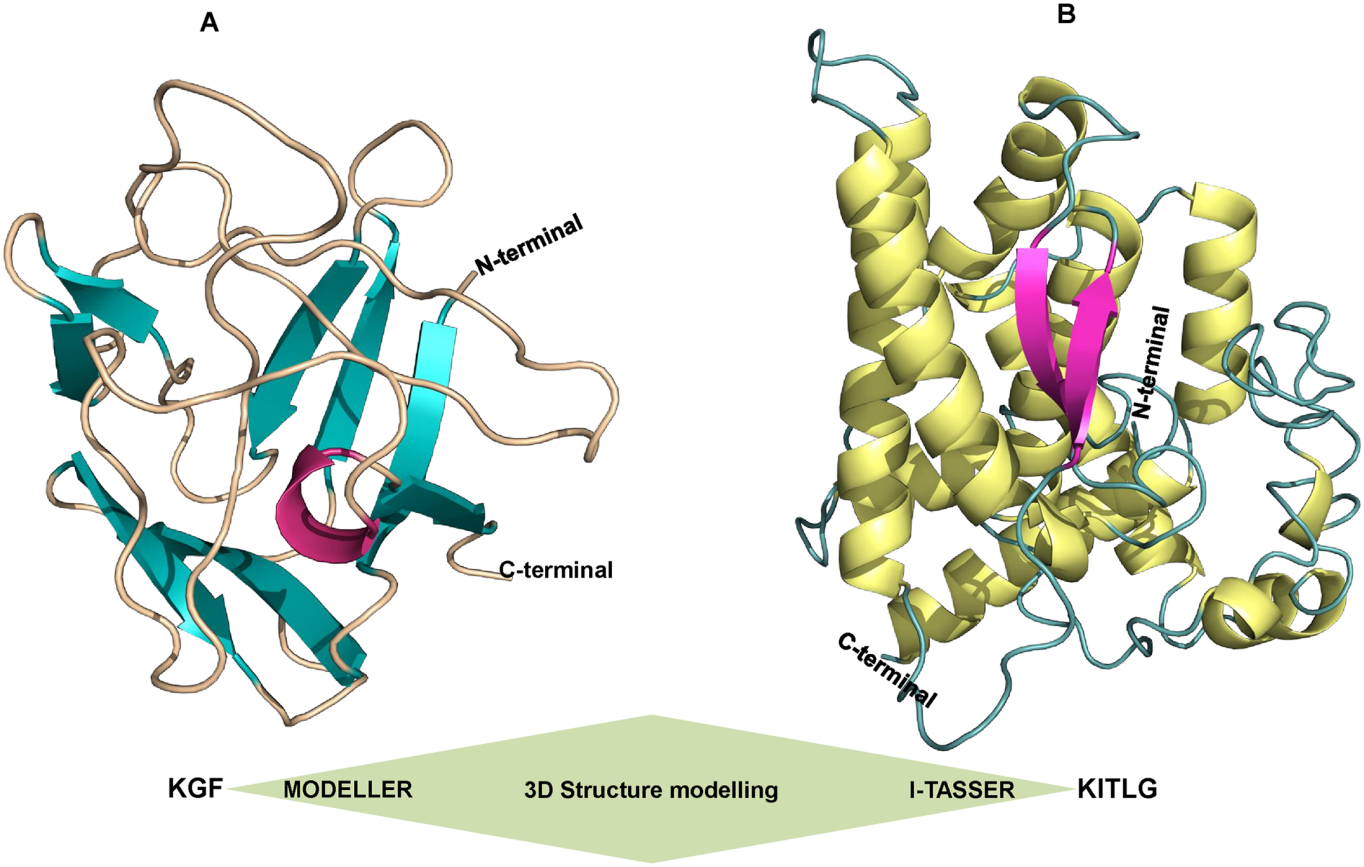
Modeling of the buffalo KGF and KITLG structures. (A) The 3D KGF protein structure generated by the MODELLER (V9.14). The N-terminus and C-terminus loops are marked. Magenta, teal and orange colors represent helix, beta sheets and loops, respectively. (B) Predicted model of KITLG by I-TASSER showing helix, beta sheets and loops in yellow, magenta and teal colors, respectively.

The 3D structures of buffalo KGF and KITLG were ascertained on the basis of homology and threading modeling methods, respectively. The tertiary structure of KGF was generated by MODELLER (V9.14) and best model selected had a DOPE score of -12370.84 and RMSD value of 0.202Å (Fig 3A). Additionally, the 3D structure of KITLG predicted employing I-TASSER server had a confidence score (C-score) of -3.39 with TM score and RMSD value of 0.34 ± 0.11 and 14.2 ± 3.8Å, respectively (Fig 3B). The structures were then submitted for energy minimization to YASARA server and returned minimized models had energies and scores of -73256.9KJ/mol; -0.86 and -117581.9KJ/mol; -2.39 for KGF and KITLG, respectively.

Geometric evaluations and stereochemical quality of the modeled 3D structures of KGF and KITLG were performed using PROCHECK by calculating the Ramachandran plot. The plot represents the distribution of phi and psi angles of the amino acid residues and classifies them in their respective quadrangle. Ramachandran plot analysis for the modeled KGF and KITLG structures showed that 95.2% and 94% residues resided in the allowed regions, respectively. Whereas, 4.0% residues in KGF and 3.2% in KITLG were present in the generously allowed regions while 0.8% of KGF and 2.8% of KITLG amino acids resided in the disallowed regions, signifying the predicted models were reliable in terms of their backbone conformation (S1 Fig). Furthermore, WHAT IF server assigned Ramachandran Z-scores of -0.409; -1.086 and structural average packing scores of -1.108; -1.006 for both KGF and KITLG models, respectively. The models were analyzed for its fold reliability using ProSA server that estimated their energy profiles (Z-score) employing molecular mechanics force field. The Z-score predicts overall model quality and measures the cumulative energy deviation of the structure using random conformations. ProSA calculated the quality score for protein structures, wherein predicted Z-scores values were -4.08 for KGF and -6.84 for KITLG, evidencing highly reliable structures. Additionally, the energy plots showed the local model quality based on plotting energies as a function of amino acid sequence position (S2 Fig). The structural error measurement at each amino acid residue in the 3D models was given by the ERRAT plot. It estimated the overall quality factor for non-bonded atom interactions and predicted ERRAT scores for KGF and KITLG were 89.92 and 92.39, respectively. This confirmed the backbone conformation and non-bonded interactions of the generated models were within the normal range (S3 Fig).

### Binding site residues and protein-protein docking

**Fig 4:**
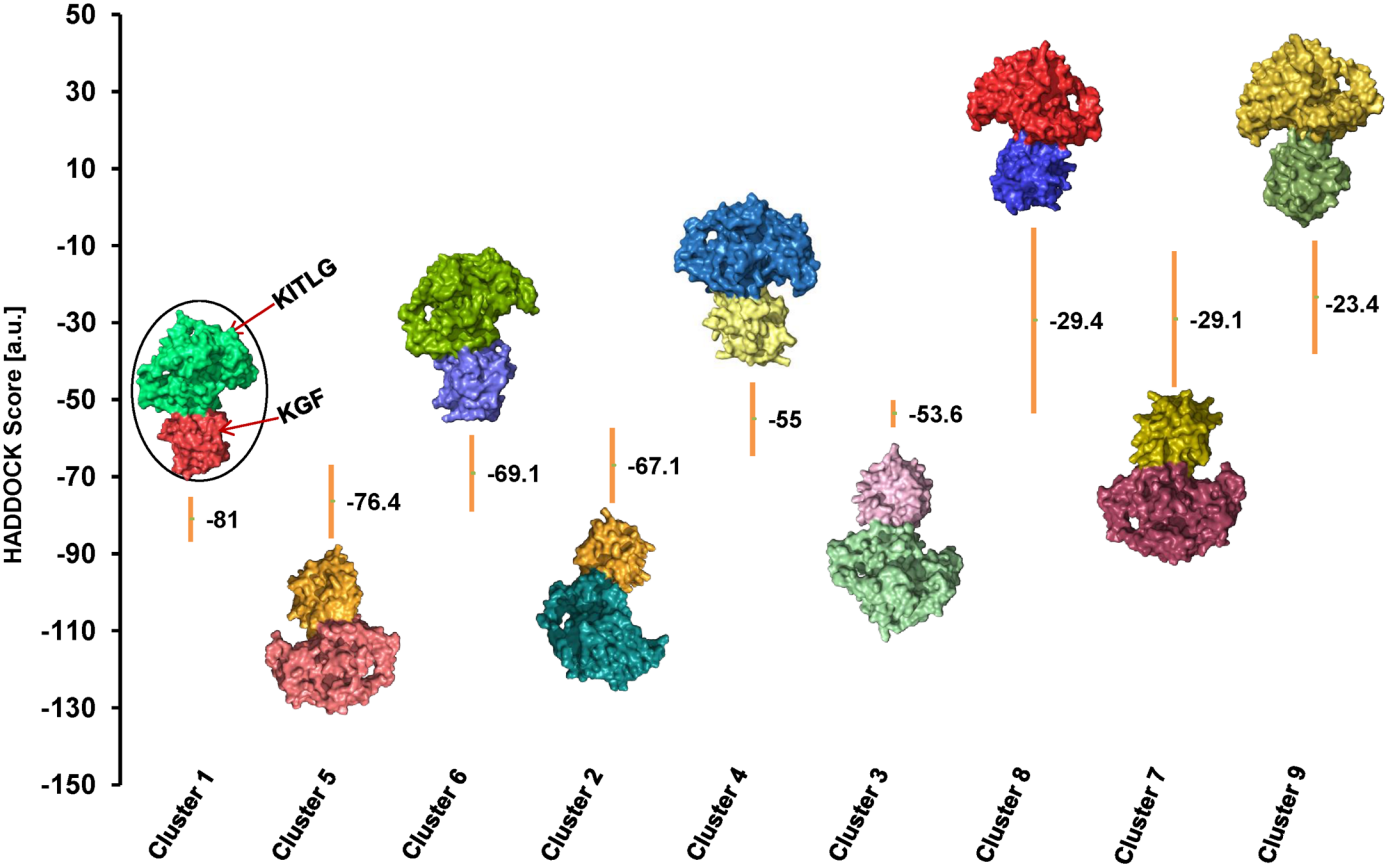
HADDOCK based structural mapping of KGF-KITLG docked complexes. HADDOCK generated 9 clusters after refinement and clustering. KGF-KITLG complexes were aligned with their respective HADDOCK scores. A surface-based protein representation in different color is used for each complex. Best docked complex (encircled) had the lowest HADDOCK score of -81.0.

**Fig 5:**
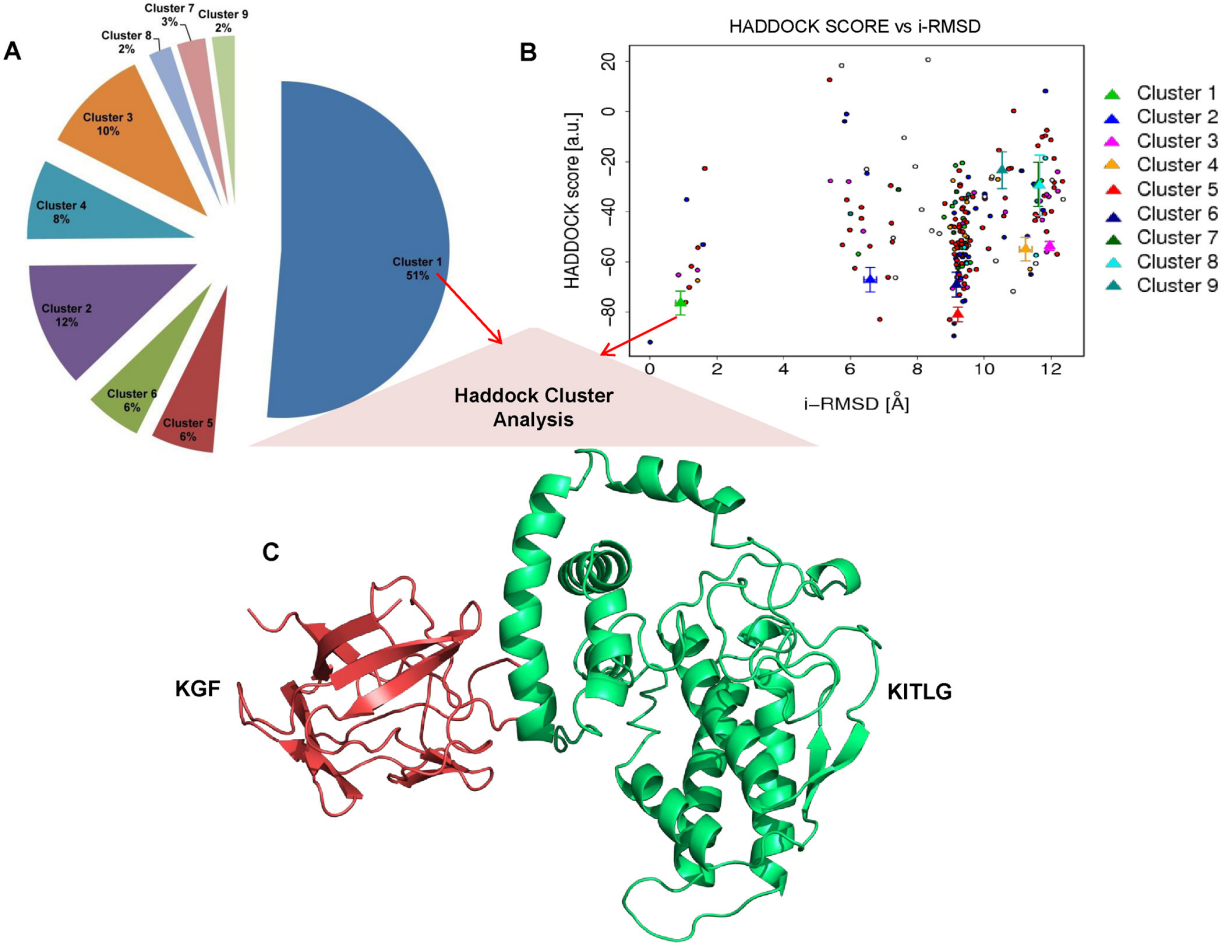
HADDOCK cluster analysis. (A) Pie-chart showing the distribution of Haddock clusters with cluster size, cluster 1 (KGF-KITLG docked complex) occupies 51% (Size—94) out of 183 complexes generated by HADDOCK (B) The HADDOCK scores of docked models were plotted against their i-RMSDs. The color codes represent the i-RMSD values of all 9 HADDOCK clusters. Wherein, cluster 1 (green) with the lowest i-RMSD value of 1.1 +/- 0.7 Å represents the best docked complex. (C) Diagrammatic illustration of selected KGF-KITLG docked complex, where KGF and KITLG are shown in red and green, respectively.

CASTp detected binding residues corresponding to KGF and KITLG proteins were subjected to protein docking (Table 2). HADDOCK clustered 183 docked complexes in 9 clusters representing 91.5% of the water-refined models (Table 3 and Fig 4). From these 9 clusters, cluster 1 with HADDOCK score:- 81.0 +/- 5.8 Kcal/mol, cluster size: 94, electrostatic energy: -222.1 +/- 19.7 Kcal/mol and Z-Score: -1.3 was selected as the best KGF-KITLG docked complex for further study (Fig 5A–5C).

**Table 2:** CASTp predictions for buffalo KGF and KITLG binding site residues.

S.No.	Protein Model	Predicted residues	Area (Å^2^)	Volume (Å^3^)
1	KGF	107 Ile, 121 Met, 122 Asn, 123 Lys, 138 Asn, 139 Phe, 140 Lys, 142 Leu, 150 Thr, 151 Tyr, 152 Ala, 153 Ser, 154 Ala, 155 Lys, 156 Trp, 157 Thr, 158 His, 159 Ser, 160 Gly, 161 Gly, 162 Glu,163 Met, 164 Phe, 181 Lys	517.2	434.5
2	KITLG	224 Phe, 225 Phe, 226 Ser, 227 Leu, 228 Val, 229 Ile, 230 Gly, 231Phe, 232 Ala, 233 Phe 234, 235 Ala, 236 Phe, 237 Tyr, 238 Trp, 240 Lys, 246 Thr, 249 Val, 250 Gly, 253 Gln, 257 Glu	193.1	213.1

**Table 3:** Statistical analysis for HADDOCK generated KGF-KITLG docked complexes.

S.No.	Cluster	HADDOCK score^a^ (a.u.)	Cluster Size	RMSD from overall lowest-energy structure (Å)	Vander Waals energy (E_vdw_) (kcal mol^-1^)	Electrostatic energy^b^ (E_elec_) (kcal mol^-1^)	Desolvation energy (E_desol_) (kcal mol^-1^)	Restraints violation energy (kcal mol^-1^)	Buried surface area (Å^2^)	Z-Score
1	1	-81.0 +/- 5.8	94	1.1 +/- 0.7	-53.5 +/- 7.0	-222.1 +/- 19.7	-1.1 +/- 6.3	181.0 +/- 35.88	1429.7 +/- 71.4	-1.3
2	5	-76.4 +/- 9.5	11	8.5 +/- 0.3	-61.9 +/- 4.7	-185.9 +/- 27.5	10.0 +/- 3.9	126.7 +/- 35.56	1811.0 +/- 95.4	-1.1
3	6	-69.1 +/- 9.9	10	9.1 +/- 0.1	-56.8 +/- 3.5	-181.0 +/- 35.0	13.7 +/- 6.9	102.0 +/- 22.82	1872.7 +/- 91.3	-0.7
4	2	-67.1 +/- 9.7	22	6.3 +/- 0.5	-50.3 +/- 5.9	-174.4 +/- 38.4	2.7 +/- 5.8	154.2 +/- 28.98	1656.2 +/- 208.9	-0.6
5	4	-55.0 +/- 9.5	14	11.7 +/- 0.5	-48.3 +/- 11.4	-163.3 +/- 48.6	12.7 +/- 5.1	133.5 +/- 34.52	1586.0 +/- 188.1	-0.1
6	3	-53.6 +/- 3.5	19	11.6 +/- 0.1	-43.9 +/- 5.7	-75.6 +/- 4.6	-4.7 +/- 8.7	100.8 +/- 30.50	1206.1 +/- 109.0	0.0
7	8	-29.4 +/- 24.1	4	11.8 +/- 0.1	-46.1 +/- 10.0	-113.1 +/- 21.8	27.1 +/- 9.1	121.4 +/- 66.72	1508.2 +/- 306.0	1.2
8	7	-29.1 +/- 17.6	5	11.4 +/- 0.1	-41.2 +/- 3.5	-127.4 +/- 35.3	23.6 +/- 9.6	140.3 +/- 67.17	1362.9 +/- 133.0	1.2
9	9	-23.4 +/- 14.7	4	9.9 +/- 0.3	-41.3 +/- 6.5	-112.6 +/- 40.2	28.2 +/- 5.7	121.1 +/- 49.24	1250.7 +/- 119.2	1.5

^a^. The ***HADDOCK score* = *E***_***vdw***_ ***+ E***_***elec***_ ***+ E***_***AIR***_

In the equation, *E*_*vdw*_ *and E*_*elec*_ represent van der Waals and electrostatic energies, respectively. Whereas, *E*_*AIR*_ indicates distance restraint contribution of AIRs.

After the water refinement, the HADDOCK score was calculated as the following weighted sum:

***HADDOCK score = 1*.*0E***_***vdw***_ ***+ 0*.*2E***_***elec***_ ***+ 1*.*0E***_***dist***_ ***+ 0*.*1E***_***solv***._ Where, *E*_*solv*_; solvation and *E*_*dist*_; distance restraints energies include both unambiguous interaction restraints and AIRs.

^b^. Non-bonded interactions were calculated with the Optimized Potentials for Liquid Simulations (OPLS) force field using 8.5Å cut-off.

### KGF-KITLG binding interface and hydrophobic interaction network

**Fig 6:**
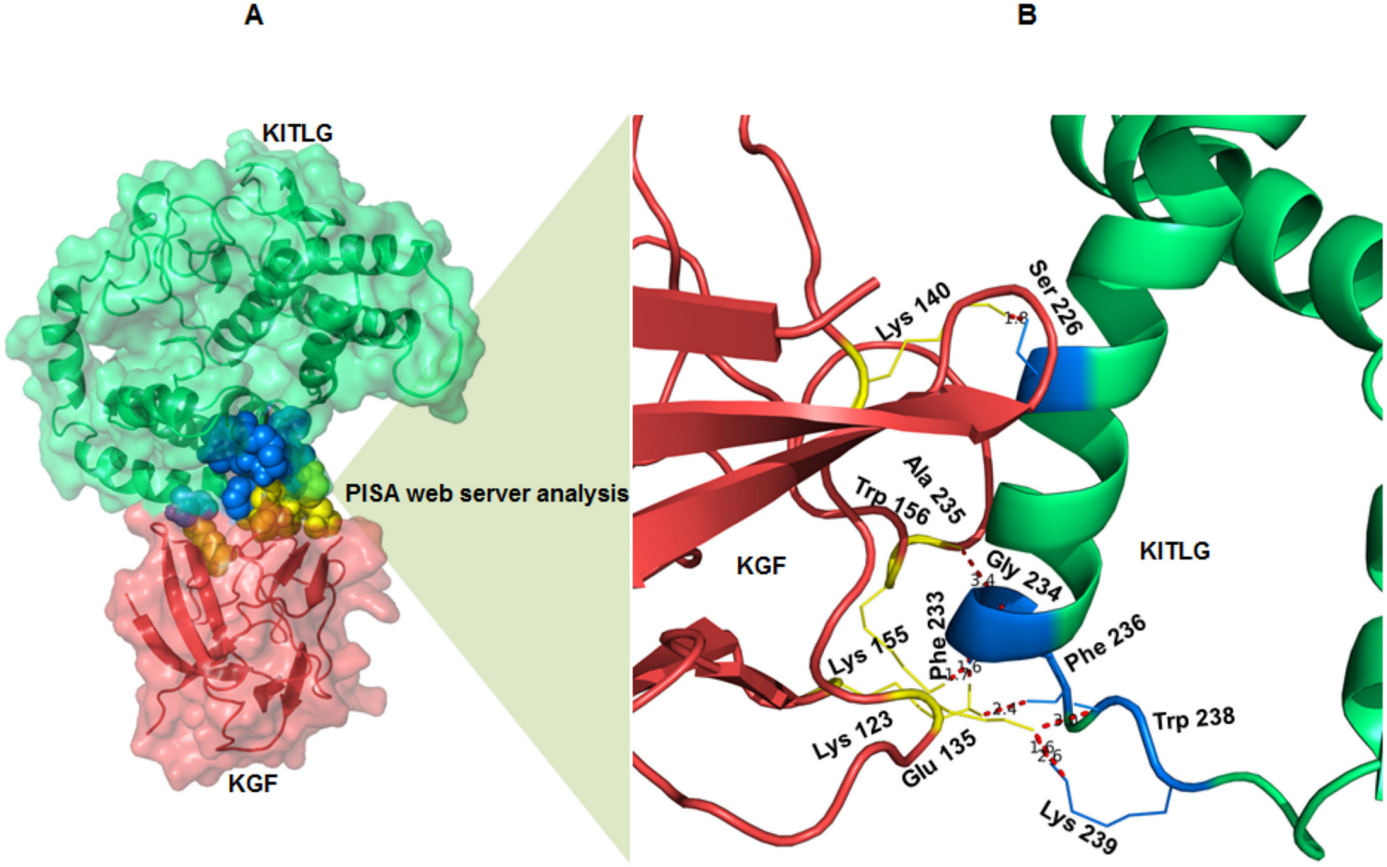
KGF-KITLG interacting interface and binding residues. (A) Structural overview of KGF-KITLG interacting interface predicted by PISA, the interacting residues are shown in spheres (KGF: yellow and KITLG: blue). (B) A close view of KGF-KITLG binding interface showing the interacting residues corresponding to KGF and KITLG proteins in yellow and blue, respectively. Dotted lines (red) represent atomic distances between hydrogen bonds formed by binding residues.

Intermolecular protein-protein interactions and surface interface areas of the docked complexes were determined using the PISA server (Table 4). KGF-KITLG complex showed interaction having an interface area of 737.6 Å^2^ and solvation free energy (Δ^i^G) as -7.5 kcal/mol. Analysis of the docked complex (KGF-KITLG) revealed the presence of residues involved in extensive H-bonding and salt bridges (Table 5). Molecular docking showed the amino acids involved in KGF-KITLG binding namely Lys123, Glu135, Lys140, Lys155, and Trp156 corresponding to KGF protein, while KITLG specific ones included Ser226, Phe233, Gly234, Ala235, Phe236, Trp238 and Lys239 (Fig 6A and 6B and Table 5). Subsequent analysis of KGF-KITLG binding interface showed the KGF interacting residues belonged to tyrosine receptor interaction site, suggesting its crucial role in regulating major activities of KGF protein through kinase activity.

**Table 4:** PISA predicted KGF- KITLG interacting interface.

KGF			KITLG			KGF-KITLG docked complex				
^i^N_at_	^i^N_res_	Surface (Å^2^)	^i^N_at_	^i^N_res_	Surface (Å^2^)	Interface area (Å^2^)	Δ^i^G (kcal/mol)	Δ^i^G P-value	N_HB_	N_SB_
63	16	7442	78	19	16303	737.6	-7.5	0.214	7	1

^i^N_at_: indicates the number of interfacing atoms

^i^N_res_: indicates the number of interfacing residues

Surface Å^2^: total solvent accessible surface area

Interface area: difference in the total accessible surface area of isolated and interfacing structures divided by 2

Δ^i^G: solvation free energy gain upon formation of the interface

Δ^i^G P-value: P-value of the observed solvation free energy gain

N_HB_: number of hydrogen bonds

N_SB_: number of salt bridges

**Table 5:** PISA analysis of the H-bonding and salt-bridge interactions among the residues participating in KGF-KITLG binding interface,

S.No.	KGF	Dist.[Å]	KITLG
Hydrogen bonds			
1	LYS 140[HZ3]	1.77	SER 226[OG]
2	LYS 155[HZ3]	1.72	PHE 233[O]
3	LYS 123[HZ2]	1.63	GLY 234[O]
4	LYS 123[HZ1]	2.37	PHE 236[O]
5	GLU 135[OE2]	3.03	TRP 238[N]
6	GLU 135[OE2]	1.57	LYS 239[HZ2]
7	TRP 156[O]	3.38	ALA 235[N]
Salt bridges			
1	Glu 135[OE2]	2.58	LYS 239[NZ]

**Fig 7:**
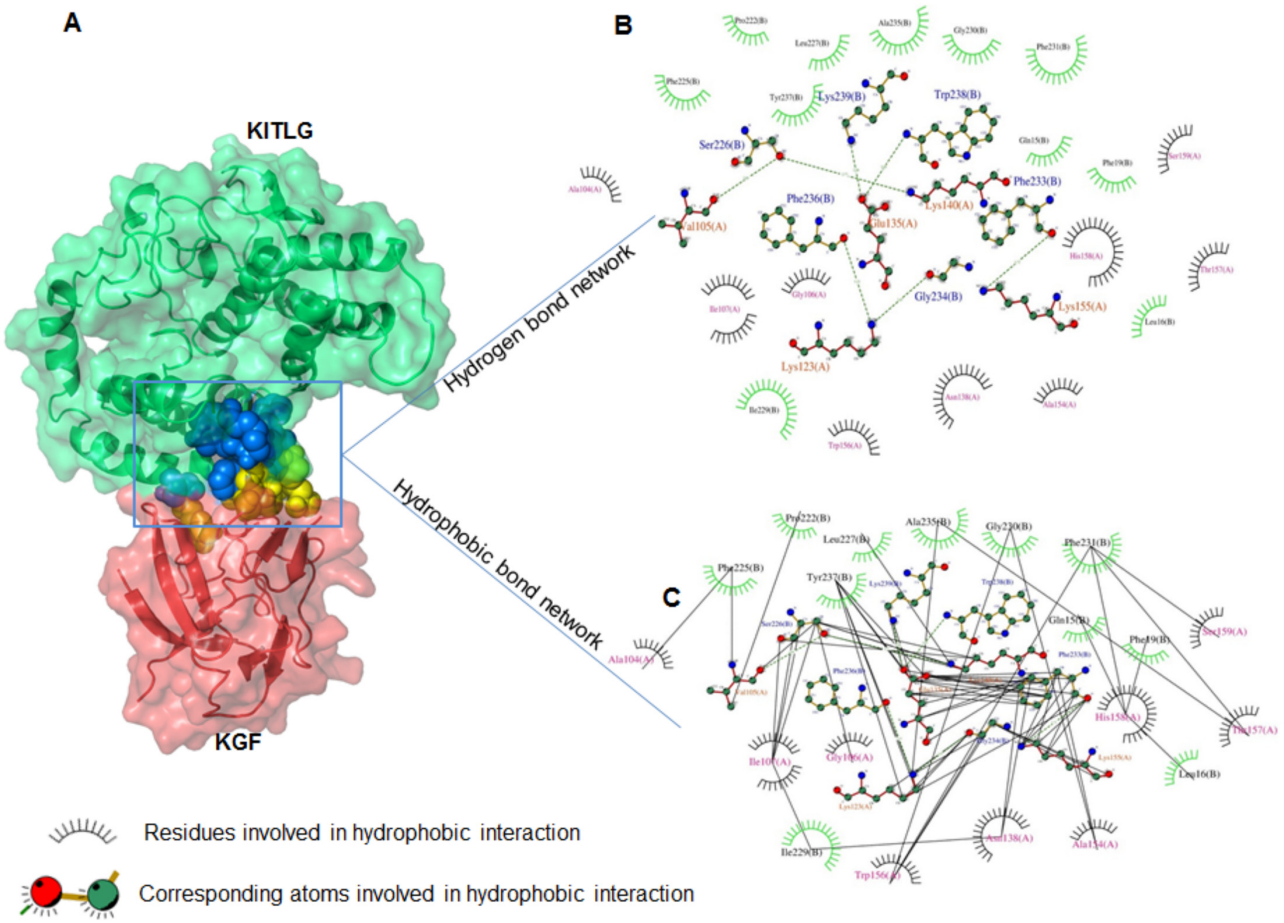
KGF-KITLG hydrophobic residual interactions. (A) KGF-KITLG docked complex with spheres representing their binding interface. (B and C) DIMPLOT program generated two-dimensional plots representing hydrogen (B) and hydrophobic (C) interactions between KGF and KITLG proteins. Green and black (dashed) lines indicate hydrogen bonds and hydrophobic interactions in KGF-KITLG complex, respectively.

Additionally, a residual network of hydrophobic interactions across the periphery of KGF-KITLG binding interface was identified using DIMPLOT program. It consists of Ala104, Gly106, Ile107, Asn138, Ala154, Trp156, Thr157, His158 and Ser159 residues belonging KGF and Gln15, Leu16, Phe19, Pro222, Phe225, Leu227, Glu230, Phe231, Ala235 and Tyr237 corresponding to KITLG protein (Fig 7A–7C). This interaction network accentuated the strong affinity and stability of KGF-KITLG binding interface.

### *In-silico* mutagenesis deviates binding energy

Bioinformatic analysis categorized the KGF mutants into two groups: group I mutants K123A, EI35A, K140A, K155A and W156A and group II mutants K123L, K140L and K155L. The comparison between the interaction energies of the mutated KGF-KITLG docked complex with that of the native one showed variation in Δ^i^G (solvation free energy gain upon formation of the interface). The Δ^i^G of group I mutants did not show much energy variation (ranging from -7.0 to -7.7 kcal/mol) as compared to that of the native complex (Δ^i^G -7.5 kcal/mol), except W156A, having the energy of -9.8 kcal/mol. Startlingly, a significant energy variation was observed in the group II mutants, especially the one where lysine residues were replaced with leucine generating a complex K140L with Δ^i^G: -11.6 kcal/mol (Table 6). The lowest value of Δ^i^G in K140L complex suggests that the mutated structure is likely to be the most stable and possibly has better binding affinity with native KITLG protein thereby, increasing the probability of its *in-vivo* occurrence.

**Table 6:** Interaction energies (Δ^i^G) of native and mutant forms of KFG-KITLG docked complex.

S.No	Protein complex	Interaction energy (Δ^i^G) (kcal/mol)
1	Native complex (KGF-KITLG)	-7.5
*Group I mutants*: *Interacting residues of KGF replaced with Alanine*		
2	K123A_KITLG	-7.0
3	E135A_KITLG	-7.6
4	K140A_KITLG	-7.7
5	K155A_KITLG	-7.7
6	W156A_KITLG	-9.8
*Group II mutants*: *Interacting Lysine residues of KGF replaced with Leucine*		
7	K123L_KITLG	-9.3
8	K140L_KITLG	-11.6
9	K155L_KITLG	-5.8

Δ^i^G indicates the solvation free energy gain upon formation of the interface and negative value corresponds to positive protein affinity.

### Similar binding interface between the KGF-KITLG homologs

All KGF-KITLG docked clusters across buffalo and cattle share a common binding interface. Within the interface, all KGF and KITLG binding residues resided from 125–185 and 220–274 amino acids, respectively (S4 Fig). This showed that KGF-KITLG homologues had conserved binding modes and common interfacial residues in both buffalo and cattle, suggesting their functional significance in regulating ovarian folliculogenesis in these species.

## Discussion

In-depth understanding of the molecular and cellular events regulating follicular development is of utmost importance to augment rate of *in vitro* fertilization. Ovarian folliculogenesis is coordinated by series of morphological, functional and regulatory intrinsic signaling pathways involving the formation of zona pellucida, proliferation of granulosa cells and active RNA synthesis within the oocyte [43–45]. Mesenchymal-derived theca cells produce a number of growth factors that act locally to regulate the proliferation of adjacent epithelial granulosa cells [5,46,47]. Considering the paracrine factors produced by the cellular compartments (granulosa/theca), the interaction between KGF and KITLG is imperative [48,49]. These protein-protein interactions seem to determine the ultimate fate and microenvironment for the maturation of ovarian follicles and granulosa cells surrounding the developing oocyte. Although in mammals, interaction of KGF and KITLG is considered to be critical for the proliferation and growth of follicular cells and oocytes during different stages, the biological requirements for this event varies across the species [49].

Several reports have shown interactions of KGF and KITLG in the ovary, using *in vitro* culture systems of rat follicles and bovine granulosa cells [6,10,50]. However, the KGF-KITLG interaction has only been studied at the transcriptional level and literature is indeed silent on their interplay at the translational level. In the present study, the co-immunoprecipitation assay followed by computational analysis confirmed the buffalo KGF and KITLG proteins interaction thereby corroborating with the earlier studies suggesting their strong interplay in ovarian folliculogenesis [9,10,49].

So far, there has been no report on the possible interacting residues responsible for KGF-KITLG interaction. We demonstrated buffalo KGF-KITLG protein interaction employing protein-protein docking approach. As crystal structures for the candidate proteins were unavailable in databases, 3D models were predicted by homology and threading modelling to deduce their functional relevance. The KGF-KITLG docked complex showed extensive hydrogen bonding optimized by hydrophobic interactions. This conferred stability to the protein structure (KGF-KITLG complex) providing specificity required for selective macromolecular interactions leading to ovarian follicles development [11,17,51–53] (S5 Fig). Thus, interaction of KGF with KITLG exhibits high degree of binding specificity for regulating its crucial biological roles.

Our *in-silico* mutagenesis observation discerned the changes in binding energy of KGF-KITLG complex, occurred on mutating the amino acids involved in their interaction. A mutated KGF-KITLG complex generated by the replacement of lysine with leucine had the lowest binding energy suggesting its high stability and likely occurrence in the *in-vivo* system. Earlier, the homology-based approach had been used for predicting the conserved intra-species PPIs with the assumption that the interaction between a pair of proteins in one species would be conserved in the other related species [54]. In the present study, we used similar approach for predicting the PPIs in the homologs of KGF and KITLG proteins in the buffalo and cattle (closely related) species. As expected, a common KGF-KITLG binding interface was detected between the two species suggesting that the binding interfaces between them are similar.

Based on the present study we construe that there exist a crosstalk between buffalo KGF and KITLG in the context of folliculogenesis. The bioinformatic based approach to understand protein-protein interaction surely complements the intricate dynamics of biological function. Therefore, further biochemical studies coupled with *in-silico* interpretations on KGF-KITLG interaction dynamics may be a rewarding proposition in providing a valuable insight into the system biology of ovarian folliculogenesis.

## Supporting Information

S1 Fig Ramachandran plot statistical analysis of KGF and KITLG models. PROCHECK derived Ramachandran evaluation plots for *Bubalus bubalis* KGF (A) and KITLG (B) 3D structures. The black dots indicate the amino acids distributed in the red (most allowed) and yellow (allowed) regions.
(TIF)

S2 Fig ProSA Z-scores of KGF and KITLG protein models. The predicted KGF and KITLG protein models had Z-scores (black point) of -4.08 and -6.84, respectively.
(TIF)

S3 Fig Validation of KGF and KITLG protein models using ERRAT. The predicted KGF (A) and KITLG (B) models yielded ERRAT scores of 89.92 and 92.39, respectively. These values indicate backbone conformation and non-bonded atomic interactions are within the acceptable range.
(TIF)

S4 Fig A pictorial representation of KGF-KITLG binding interface corresponding to buffalo and cattle species. (A) Interacting domain view of KGF and KITLG proteins. KGF-KITLG docked complex showed the common interacting domains between the two species. Wherein, the binding residues ranged from 125–185 and 220–270 in KGF and KITLG, respectively. (B) KGF-KITLG docked complex for buffalo and cattle showing KGF (magenta) and KITLG (yellow) interacting residues. The protein chains are shown in stick representation. (C) Superimposed model of KGF-KITLG complex of buffalo and cattle representing conserved binding interface in both species.
(TIF)

S5 Fig Diagrammatic illustration of KGF-KITLG interactions during different stages of ovarian folliculogenesis. Proposed schematic model represents cell-cell interactions during follicular development. Predicted 3D models of buffalo KGF and KITLG proteins and their complex were generated by protein docking (encircled). KGF-KITLG complex implicated in different stages of follicular development is shown in blue background. Arrows indicate the potential targets of KGF-KITLG complex, where ‘+’ headed ones denote growth at the respective developmental stages.
* Basic fibroblast growth factor-bFGF; Leukemia inhibitory factor- LIF.
(TIF)
