# Supplementary figures and images for: Cross Talk between KGF and KITLG Proteins Implicated with Ovarian Folliculogenesis in Buffalo Bubalus bubalis
Source: PLoS One. 2015 Jun 17;10(6):e0127993. doi: 10.1371/journal.pone.0127993 (PMC4470682; doi:10.1371/journal.pone.0127993)

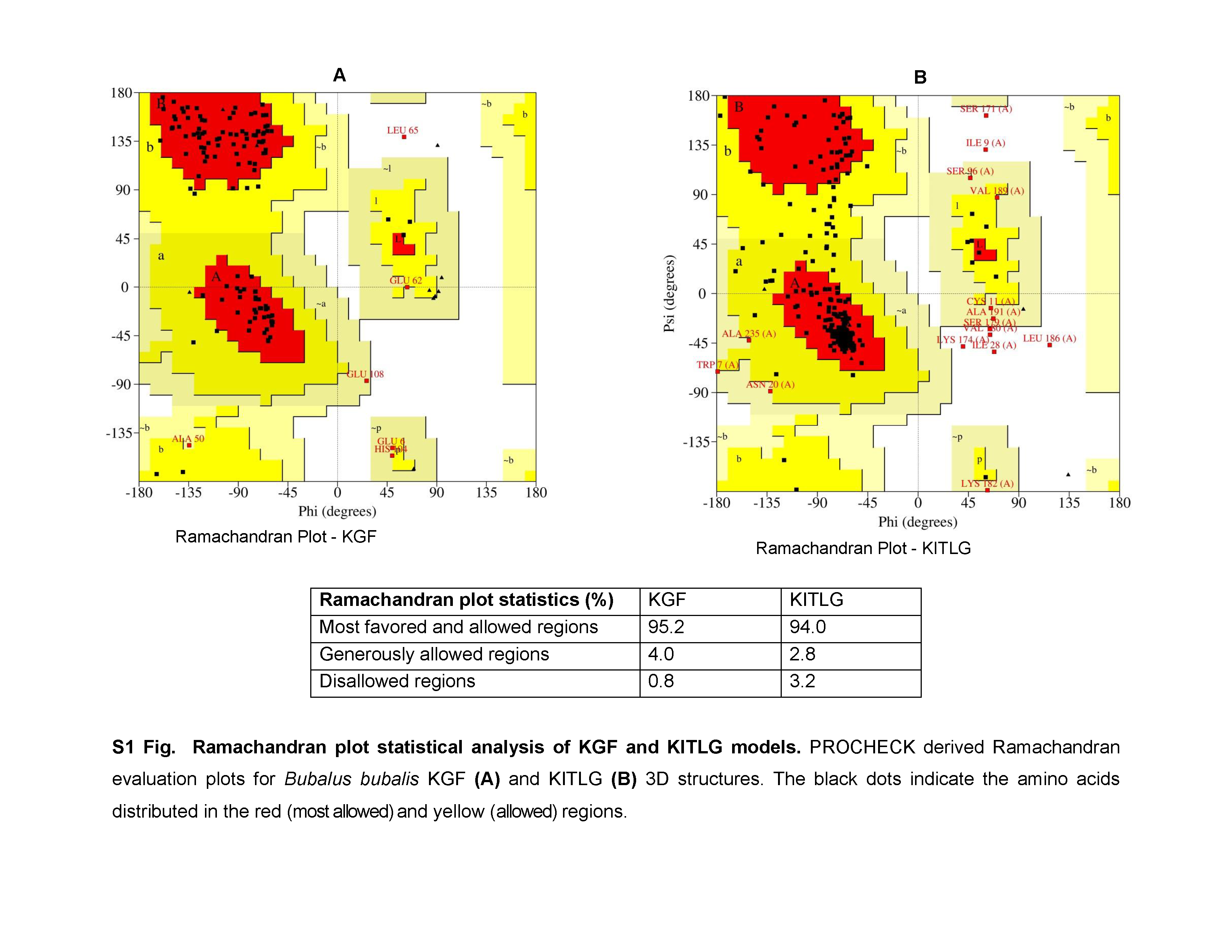

Supplement: S1 Fig — PROCHECK derived Ramachandran evaluation plots for Bubalus bubalis KGF (A) and KITLG (B) 3D structures. The black dots indicate the amino acids distributed in the red (most allowed) and yellow (allowed) regions. (TIF) [file pone.0127993.s001.tif]

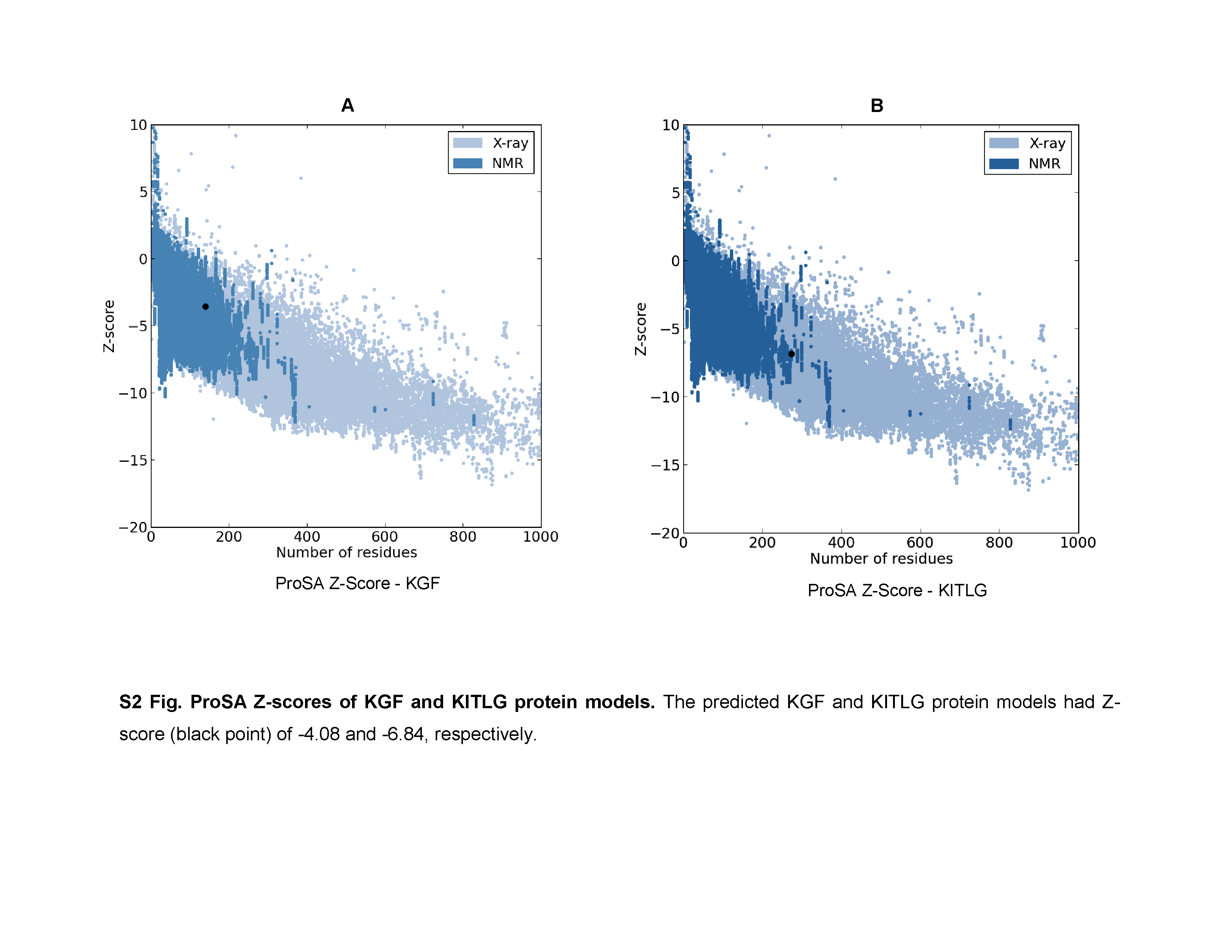

Supplement: S2 Fig — The predicted KGF and KITLG protein models had Z-scores (black point) of -4.08 and -6.84, respectively. (TIF) [file pone.0127993.s002.tif]

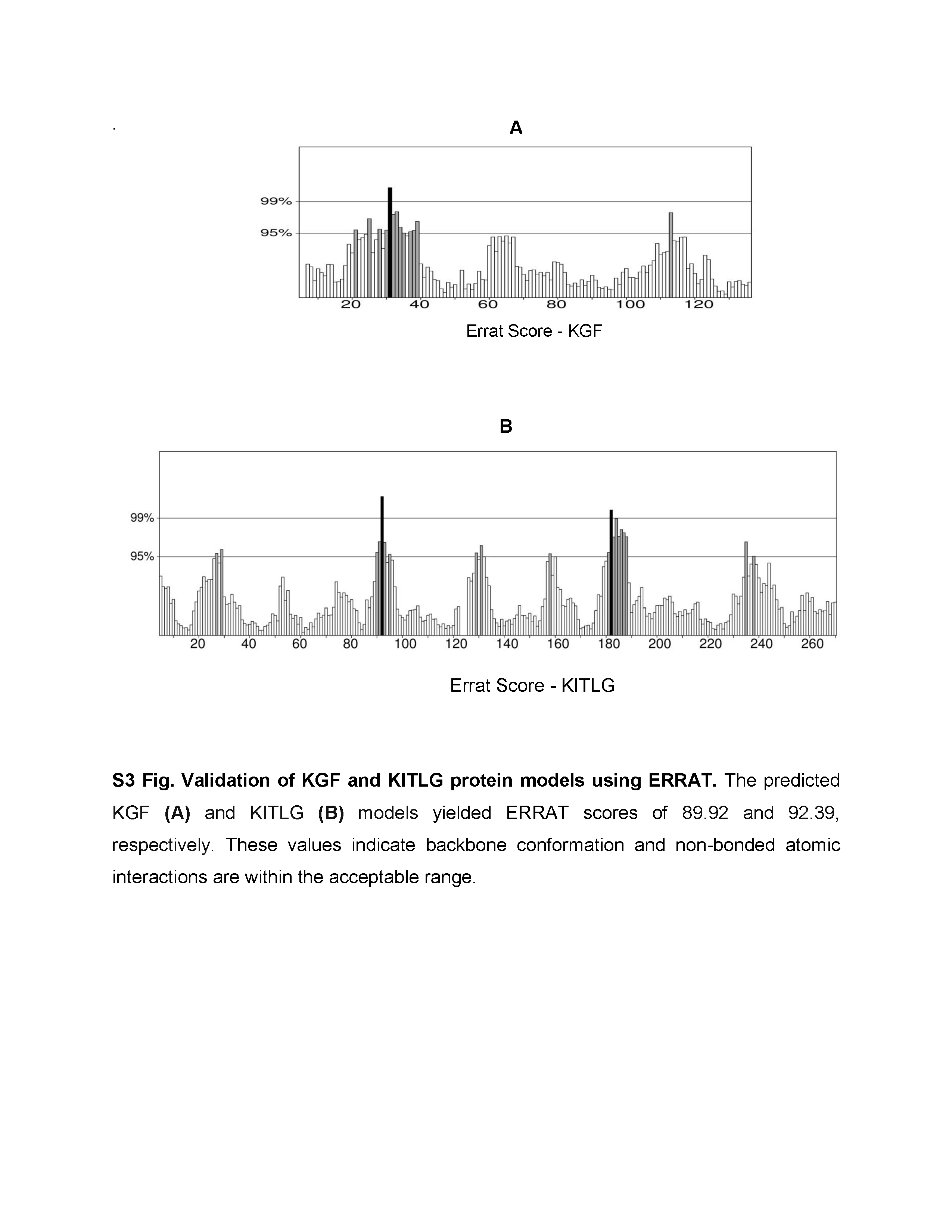

Supplement: S3 Fig — The predicted KGF (A) and KITLG (B) models yielded ERRAT scores of 89.92 and 92.39, respectively. These values indicate backbone conformation and non-bonded atomic interactions are within the acceptable range. (TIF) [file pone.0127993.s003.tif]

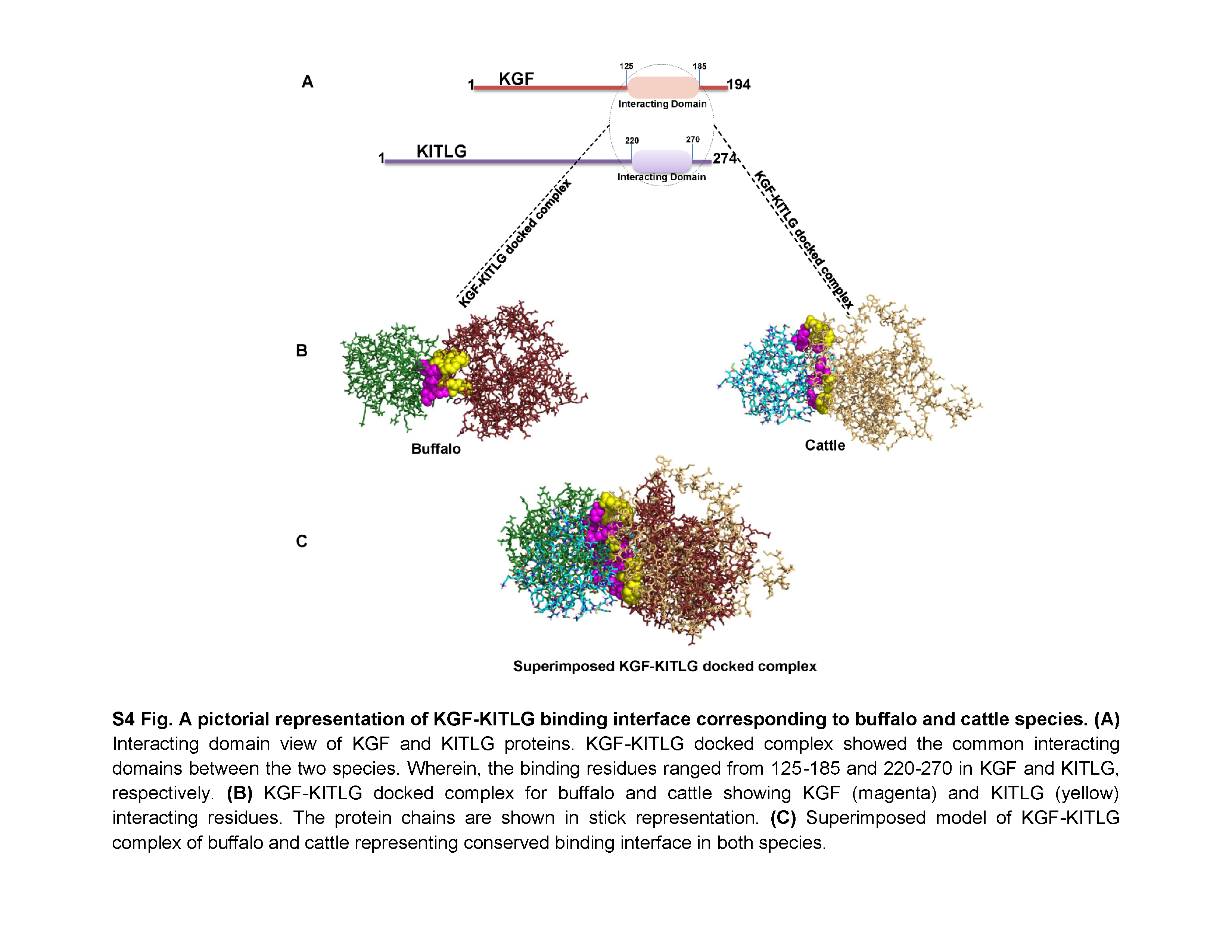

Supplement: S4 Fig — (A) Interacting domain view of KGF and KITLG proteins. KGF-KITLG docked complex showed the common interacting domains between the two species. Wherein, the binding residues ranged from 125–185 and 220–270 in KGF and KITLG, respectively. (B) KGF-KITLG docked complex for buffalo and cattle showing KGF (magenta) and KITLG (yellow) interacting residues. The protein chains are shown in stick representation. (C) Superimposed model of KGF-KITLG complex of buffalo and cattle representing conserved binding interface in both species. (TIF) [file pone.0127993.s004.tif]

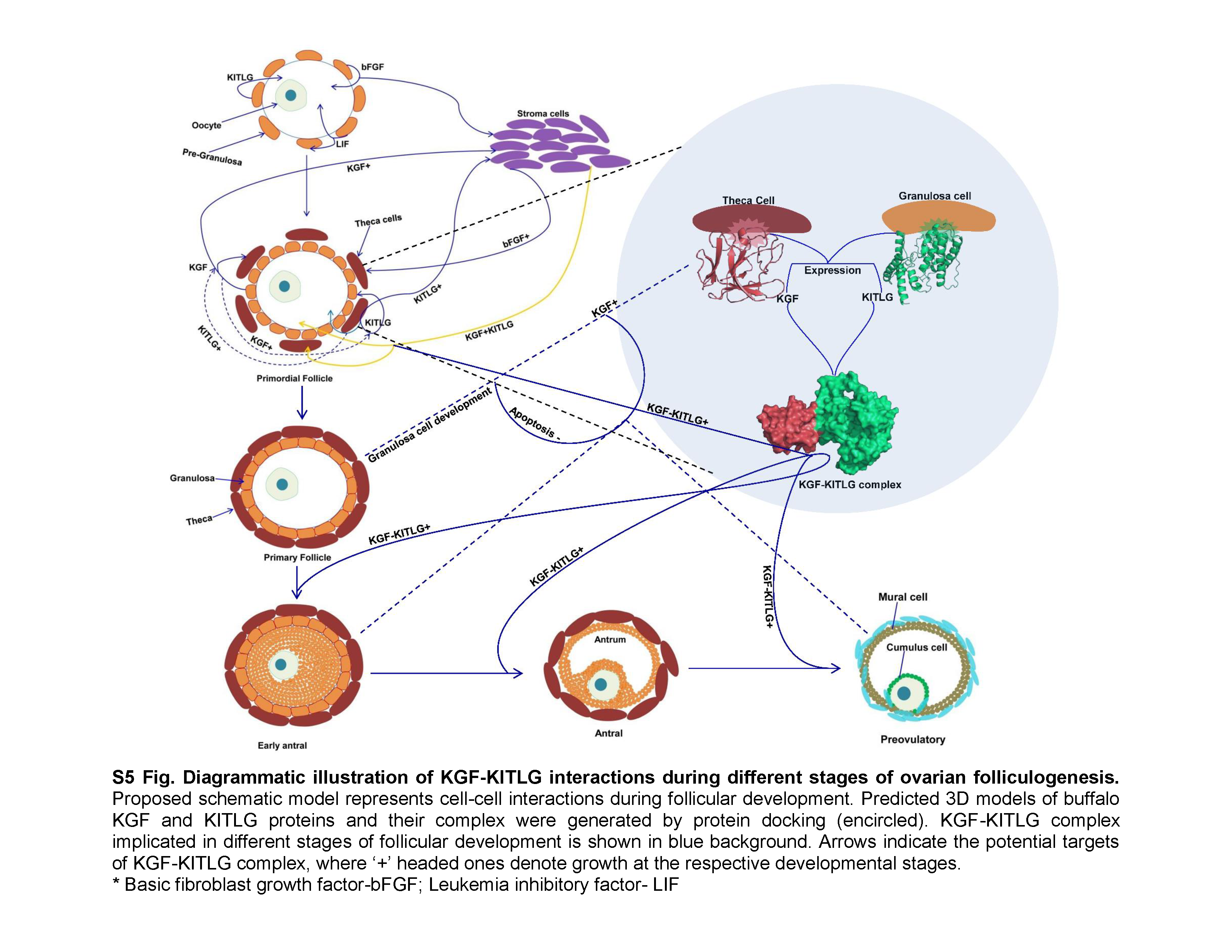

Supplement: S5 Fig — Proposed schematic model represents cell-cell interactions during follicular development. Predicted 3D models of buffalo KGF and KITLG proteins and their complex were generated by protein docking (encircled). KGF-KITLG complex implicated in different stages of follicular development is shown in blue background. Arrows indicate the potential targets of KGF-KITLG complex, where ‘+’ headed ones denote growth at the respective developmental stages. * Basic fibroblast growth factor-bFGF; Leukemia inhibitory factor- LIF. (TIF) [file pone.0127993.s005.tif]
